# Markers of endothelial pathology to support detection of atrial fibrillation in embolic stroke of undetermined source

**DOI:** 10.1038/s41598-019-55943-9

**Published:** 2019-12-19

**Authors:** Nora L. Ziegler, Jan-Thorben Sieweke, Saskia Biber, Maria M. Gabriel, Ramona Schuppner, Hans Worthmann, Jens Martens-Lobenhoffer, Ralf Lichtinghagen, Stefanie M. Bode-Böger, Udo Bavendiek, Karin Weissenborn, Gerrit M. Grosse

**Affiliations:** 1 Department of Neurology, Hannover Medical School, Hannover, Germany; 2 Department of Cardiology and Angiology, Hannover Medical School, Hannover, Germany; 3 Institute of Clinical Pharmacology, Otto-von-Guericke University Magdeburg, Magdeburg, Germany; 4 Institute of Clinical Chemistry, Hannover Medical School, Hannover, Germany

**Keywords:** Diagnostic markers, Atrial fibrillation, Stroke, Embolism, Stroke

## Abstract

A relevant part of embolic strokes of undetermined source (ESUS) is assumed to be cardiogenic. As shown previously, certain biomarkers of endothelial pathology are related to atrial fibrillation (AF). In this long-term follow-up study, we aimed to investigate whether these biomarkers are associated with subsequently diagnosed AF and with atrial cardiopathy. In 98 patients who suffered ischemic stroke of known and unknown origin L-arginine, Asymmetric (ADMA) and Symmetric Dimethylarginine (SDMA) have been measured on follow-up at least one year after index stroke. Stroke-diagnostics were available for all patients, including carotid Intima-Media-Thickness (CIMT) and comprehensive echocardiography studies. CIMT was larger in AF- compared with ESUS-patients (P < 0.001), independently from CHA_2_DS_2_VASC in the regression analysis (P = 0.004). SDMA-values were stable over time (P < 0.001; r = 0.788), whereas for ADMA moderate correlation with the initial values could be found (P = 0.007; r = 0.356). According to Kaplan-Meier-analyses, AF-detection rates were associated with CIMT (P = 0.003) and SDMA (P < 0.001). SDMA correlated with left atrial volume-index within the whole collective (P = 0.003, r = 0.322) and within the ESUS-subgroup (P = 0.003; r = 0.446). These associations were independent from CHA_2_DS_2_VASC and renal function in the regression analysis (P = 0.02 and P = 0.005, respectively). In conclusion, these results highlight SDMA and CIMT as potential markers of atrial cardiopathy and AF in ESUS-patients.

## Introduction

Approximately 25% of all ischemic strokes cannot be attributed to any cause. However, the determination of stroke etiology is crucial for its secondary prevention. Hart *et al*. introduced the concept of “embolic stroke of undetermined source” (ESUS) in 2014^1^. ESUS is presumed of being cardioembolic in a significant proportion. Consecutively, the two trials NAVIGATE-ESUS^2^ and RESPECT-ESUS^3^ have been initiated but finally failed to demonstrate a superiority of anticoagulation compared to anti-platelet therapy with acetylsalicylic acid in secondary prevention after ESUS^4,5^. Thus, novel markers supporting the diagnosis of stroke etiology in ESUS are highly warranted for the purpose of choosing the appropriate therapy.

Current pathophysiological concepts of cardioembolism describe a systemic disorder which is associated with enlargement and further remodeling mechanisms of the left atrium, currently referred as atrial cardiopathy^6^. Presumably, atrial cardiopathy leads to enhanced thrombembolic risk even beyond occurrence of atrial fibrillation (AF) which may be interpreted as a symptom at the end stage of this chronic process^7^.

Mechanisms of endothelial dysfunction are closely linked to the development of atrial cardiopathy^8,9^. In a previous study investigating novel echocardiographic and blood-based biomarkers for detection of AF^10^, we therefore addressed endothelial (dys)function markers, i.e. asymmetric dimethylarginine (ADMA), symmetric dimethylarginine (SDMA) as well as L-arginine, as potential parameters for individual risk stratification for AF in patients with embolic stroke^11^. As representative of advanced stages of endothelial damage the Carotid Intima-Media-Thickness (CIMT) was considered. Importantly, our results demonstrated that especially values of SDMA and CIMT were significantly altered in AF-patients compared to those with ESUS^11^. Of note, SDMA, in contrast to ADMA, has been previously associated with left atrial dimension in the general population^12^ and was described of being downregulated after left atrial appendage closure^13^, implicating a causal relation with atrial cardiopathy.

In this study, we aimed to investigate the role of the described markers of endothelial pathology as indicators of atrial cardiopathy and fibrillation in ESUS. The patients considered in our previous work have been followed up at least one year after index stroke to investigate the long-term temporal pattern of the biomarker levels and to search for associations with subsequently diagnosed AF and with echocardiographic parameters of the left atrium. We sought to contribute to a more precise classification of the individual risk for atrial cardiopathy and atrial fibrillation in ESUS on a biomarker level.

## Results

### Epidemiological data

Of 121 patients being enrolled in the previous study^10^ 103 individuals were available for a complete long-term follow-up examination between August 2017 and April 2018. Five participants were excluded subsequently because of evidence of newly diagnosed malignant disease in the follow-up period or identification of a rare stroke mechanism as described below, leaving 98 patients for complete analysis. In 10 of these patients the follow-up investigation could be performed only via telephone, which results in the absence of NIHSS and blood collection for these subjects. Three of the considered 103 patients died within the follow-up period. See Figure S1 in the online supplement for detailed information.

Table 1 provides the epidemiological data of the study population at the end of follow-up. The study cohort included the following groups: 45 patients with ESUS, 23 patients with AF including 18 patients who were newly diagnosed with AF within the whole study period, 11 patients with stroke due to macro- and 19 patients with stroke due to microangiopathy.

**Table 1:** Epidemiological and clinical characteristics of the study collective.

N	ESUS	AF		Macroangiopathic stroke	Microangiopathic stroke	P-value
		Previously diagnosed	Newly diagnosed			
	45	5	18	11	19	
Age (a) (IQR)	69.00^$§§^ (53.00–76.50)	80.00*^ß^ (74.50–89.00)	79.00**^ß^ (75.75–82.25)	69.00^$§^ (63.00–75.00)	68.00 (63.00–81.00)	0.004
Sex (male)	27 (60.0%)	2 (40.0%)	14 (77.8%)	7 (63.6%)	11 (57.9%)	0.532
BMI (kg/m²) (IQR)	26.73 (23.08–28.69)	25.59 (21.07–26.01)	26.24 (25.00–30.38)	23.15^#^ (21.46–27.76)	27.13^ß^ (25.01–33.81)	0.119
ESRS (IQR)	3.0^§§^ (2.0–4.0)	4.0 (4.0–4.0)	4.5**^#^ (4.0–5.0)	3.0 (2.0–5.0)	3.0^§^ (3.0–5.0)	0.027
CHA_2_DS_2_VASC (IQR)	4.0^§§^ (3.0–5.0)	5.0 (4.5–6.0)	5.5**^#^ (5.0–6.0)	5.0 (4.0–6.0)	4.0^§^ (4.0–6.0)	0.007
NIHSS on admission (IQR)	2.0 (1.0–5.0)	3.0 (0–13.0)	2.0^#^ (1.0–3.25)	3.0 (1.0–4.0)	3.0^§^ (2.0–5.0)	0.305
NIHSS on follow-up (IQR)	0 (0–1.0)	0 (0–2.0)	0 (0–1.0)	0 (0–12.0)	0 (0–2.0)	0.859
mRS on follow-up (IQR)	1.0 (0–1.5)	1.0 (0–2.0)	1.0 (0–3.0)	1.0 (0–4.0)	1.0 (0–4.0)	0.364
Barthel-Index (IQR)	100 (97.50–100)	95 (87.50–100)	100 (93.75–100)	100 (40–100)	100 (50–100)	0.491
Symptom to venous puncture time on follow-up (d) (IQR)	391.50 (372.75–453.50)	401 (387.50–437.00)	379 (375.25–421.00)	374.50 (371.25–443.75)	378 (366.50–423.50)	0.463
eGFR (ml/min/1.73 m²) (IQR)	81^§^ (66.08–100.04)	63.50 (54.91–86.21)	70.46*^#^ (57.39–80.90)	72.00 (61–101.00)	81.00^§^ (72.47–93.00)	0.156
Serum creatinine (µmol/l) (IQR)	79.00 (66.50–94.00)	75.00 (66.00–97.00)	89.00 (74.00–105.25)	95.00 (64.00–103.00)	77.00 (72.00–86.00)	0.352
Cumulative time of Holter-ECG (h) (IQR)	72.00^#^ (70.00–93.05) (N = 45)	47.50 (24.00) (N = 2)	70.00 (46.50–76.00) (N = 16)	72.00 (48.00–96.00) (N = 11)	72.00* (48.00–72.00) (N = 19)	0.071

The Essen Stroke Risk Score (ESRS) subsumes following risk factors: arterial hypertension, diabetes mellitus, age, previous myocardial infarction, previous stroke or transient ischemic attack, peripheral arterial disease, other cardiovascular disease and nicotine abuse. The CHA_2_DS_2_VASC subsumes the risk factors congestive heart failure, arterial hypertension, age, diabetes mellitus, history of stroke or transient ischemic attack or thromboembolism, other vascular disease and sex. ^*/**^ indicates significant differences with the ESUS-group. ^$/$$^ indicates significant differences with the previously diagnosed AF group. ^§/§§^ indicates significant differences with the newly diagnosed AF group. ^ß/ßß^ indicates significant differences with the macroangiopathic stroke group. ^#/##^ indicates significant differences with the microangiopathic stroke group. Values were calculated using the Kruskal-Wallis-test and chi-square test. A p-value < 0.05 was considered significant.

There were significant differences between the groups regarding the following baseline parameters (Table 1): age, BMI, ESRS, CHA_2_DS_2_VASC and eGFR. The median time to follow-up was 386 days (range: 359d-599d) and did not significantly differ between the study groups. For further information on secondary stroke preventive therapy and recurrent ischemic events see the online supplement.

### Cardiac monitoring and detection of AF during the follow-up period

In five of 23 patients with confirmed AF, this diagnosis was previously known. In 18 patients AF was newly diagnosed during in hospital-stay via bedside monitoring or in Holter-ECG in the course of the previous study (n = 12) or within the one-year-follow-up period (n = 6), respectively. Of the 6 patients with newly diagnosed AF within the follow-up period, three patients were previously assigned to the ESUS-group, two to the group with macroangiopathic stroke and one to the group with microangiopathic stroke.

Additional cardiac monitoring using Holter-ECG in the follow-up period was performed in 29 patients of the whole study collective, which resulted in a new diagnosis of AF in two cases (6.9%). Furthermore, AF was diagnosed in three subjects in the course of a new hospital admission, either in the standard admission ECG or in subsequent stroke monitoring. In one patient AF was found via regular check-up ECG. Three patients of the whole study collective were provided with cardiac event recorders within the follow up period. In these, no AF has been detected within the study period.

The cumulative time of Holter-ECG recording was 72 h in median for the whole collective, as well as for the ESUS-subgroup. The distribution of the cumulative time of rhythm analyses within the study groups is presented in Table 1.

### L-arginine and dimethylarginines in long-term follow-up after stroke

**Figure 1:**
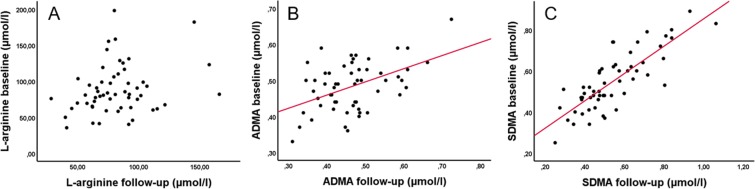
Levels of L-arginine (**A**), ADMA (**B**) and SDMA (**C**) at baseline (7 days) and follow-up. Spearman correlation indicates a significant positive correlation for ADMA (r = 0.356, P = 0.007) and SDMA (r = 0.788, P < 0.001).

L-arginine and dimethylarginine levels were available for 63 patients at baseline and for 87 on follow-up. Comparison of the follow-up values with baseline values revealed that SDMA-levels were highly stable over time according to Spearman correlation (r = 0.788, P < 0.001, Fig. 1C). Also, ADMA-follow-up-levels correlated with the according baseline values (r = 0.356, P = 0.007, Fig. 1B), while there was no significant correlation regarding L-arginine levels between the two time points (r = 0.209, P = 0.121, Fig. 1A). According correlations were calculated for ADMA:SDMA-ratio (r = 0.647, P < 0.001), L-arginine:SDMA-ratio (r = 0.571, P < 0.001) and L-arginine:ADMA-ratio (r = 0.294, P = 0.028). There was no association of changes of dimethylarginine levels over time with stroke severity, previous thrombolytic therapy or study groups (data not shown).

SDMA levels at follow-up were significantly higher in AF- than in ESUS-patients (P = 0.047). The binary regression analysis revealed that SDMA was not independently different between ESUS and AF when considering ESRS or CHA_2_DS_2_VASC.

**Figure 2:**
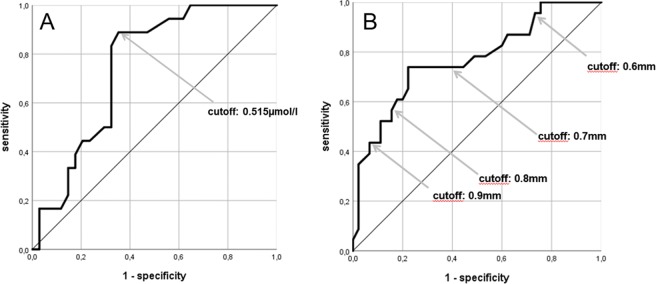
(**A**) ROC-analysis for distinguishing AF from ESUS with SDMA baseline values. The arrow indicates a cutoff level at 0.515 µmol/l; (**B**) ROC-analysis for distinguishing AF from ESUS using CIMT. Arrows indicate various CIMT-cutoffs (0.6 mm–0.8 mm).

**Figure 3:**
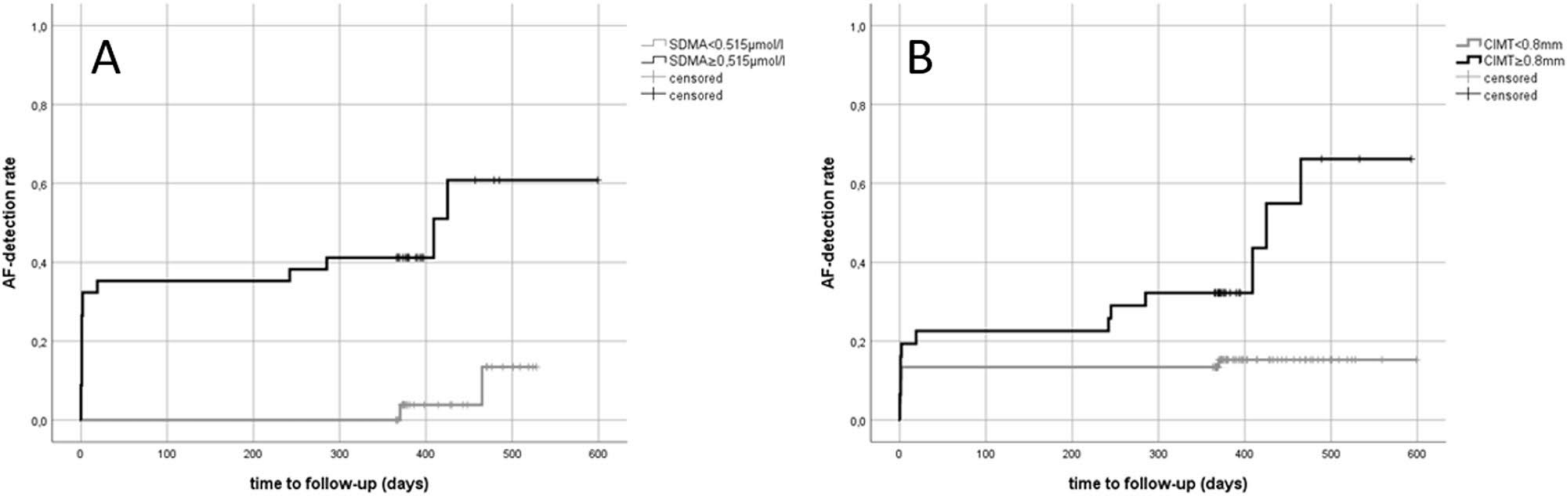
(**A**) Inverse Kaplan-Meier curves comparing AF detection rates over time between patients with baseline SDMA values <0.515 µmol/l (n = 24) and ≥0.515 µmol/l (n = 23); (**B**) Inverse Kaplan-Meier curves comparing AF detection rates over time between patients with CIMT <0.8 mm (n = 67) and ≥0.8 mm (n = 31).

The ROC-analysis showed an area under the curve (AUC) of 0.654 (95% CI: 0.512–0.796) regarding the follow-up SDMA values for distinguishing AF from ESUS. Regarding baseline SDMA levels the according ROC-analysis revealed an AUC of 0.746 (P = 0.004; 95% CI: 0.614–0.878). The optimal SDMA cutoff according to the Youden-index was identified at 0.515 µmol/l (P = 0.002; AUC: 0.768, 95% CI: 0.635–0.901) (Fig. 2A). When using this cutoff in Kaplan-Meier analysis, there were distinct AF-detection rates over time which were significantly different according to the log-rank test in the whole study cohort (P < 0.001) (Fig. 3A).

### Dimethylarginines and echocardiographic markers of atrial cardiopathy

**Figure 4:**
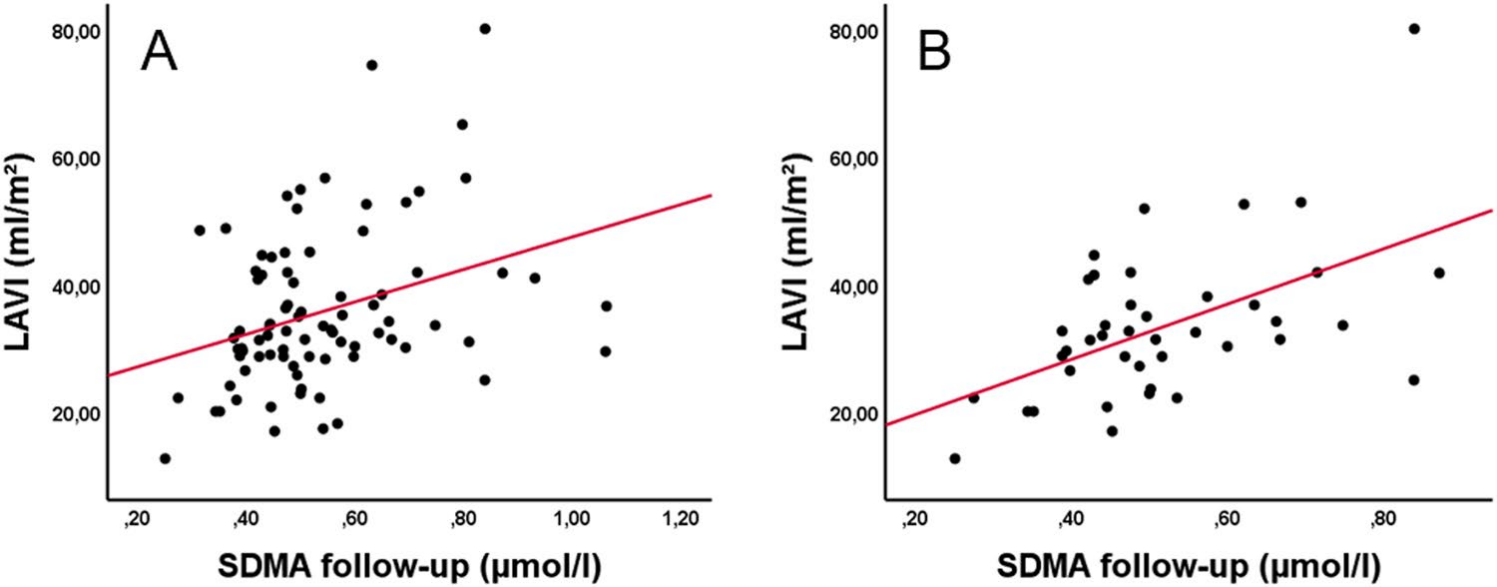
Spearman correlation of LAVI and SDMA levels on follow-up in the whole study collective (r = 0.322, P = 0.003) (**A**) and the ESUS-subgroup (r = 0.446, P = 0.003) (**B**).

Transthoracic echocardiography was performed in 97 patients, 49 of these underwent additional transesophageal echocardiography. Within the total study collective, LAVI significantly correlated with follow-up levels of ADMA and SDMA (Fig. 4A) (P < 0.001, P = 0.003, respectively), which passed level of significance after Bonferroni-correction. In a linear regression analysis considering CHA_2_DS_2_VASC and eGFR, SDMA proved to be independently associated with LAVI (P = 0.02), while ADMA did not (P = 0.054).

Within the ESUS-subgroup, SDMA levels at follow-up also significantly correlated with LAVI (P = 0.003) (Fig. 4B). In the according linear regression analysis SDMA again proved to be independently associated with LAVI (P = 0.005) in ESUS-patients. Furthermore, SDMA positively correlated with lateral PA-TDI (P = 0.005). However, this association did not pass the level of significance in the linear regression analysis (P = 0.061). Supplemental Tables II and III provide an overview of the associations between biomarkers and echocardiography.

### Carotid intima-media thickness as an independent factor for distinguishing ESUS and AF

Patients with known and newly diagnosed AF, as well as those with macroangiopathic stroke, showed significantly increased CIMT values compared to ESUS-patients (P = 0.001, P < 0.001, P = 0.024, respectively). In the binary logistic regression, including CHA_2_DS_2_VASC, CIMT proved to be independently different between AF and ESUS (P = 0.004). This difference was confirmed in the regression analysis comparing ESUS- with newly diagnosed AF-patients (P = 0.004).

ROC-analyses revealed a sufficient discriminability between ESUS and AF in total (Fig. 2B) as well as between ESUS and newly diagnosed AF (AUC = 0.77 (95% CI: 0.647–0.892), P < 0.001; AUC = 0.804 (95% CI: 0.682–0.925), P < 0.001, respectively). For CIMT cutoffs and according levels of sensitivity and specificity see Fig. 2B. The Kaplan-Meier analysis showed significantly different AF-detection rates between patients with CIMT values below compared to those above 0.8 mm according to the log-rank test in the whole sample (P = 0.003) (Fig. 3B).

### Markers of endothelial pathology and thrombembolic risk

For associations between markers of endothelial pathology and thrombembolic risk scores as well as recurrent ischemic events see the online supplement.

## Discussion

Given the current evidence from the clinical trials RESPECT-ESUS^5^ and NAVIGATE-ESUS^4^ novel biomarkers indicating AF and atrial cardiopathy in patients who suffered ESUS are of high interest. Besides blood-biomarkers, parameters based on ECG-analyses for the detection of atrial cardiopathy have been previously introduced, like e.g. the P-wave terminal force in ECG lead V1 (PTFV1)^14,15^ or RR-interval^16^. Interestingly, ECG-based biomarkers were only weakly associated with occurrence of AF after ESUS^17^. The N-terminal fragment of the brain natriuretic peptide (NT-proBNP), however, is an established marker of heart failure and was repeatedly considered being predictive for AF^18–20^. Even so, there is also evidence that NT-proBNP values are altered due to ischemic stroke itself, depending on lesion size^21,22^, which may be a limiting factor regarding diagnostic application. As a result, additional markers supporting the detection of AF in ESUS patients would be beneficial.

### Dimethylarginines on long-term follow-up

As previously described^11^, SDMA but not ADMA levels are associated with detection of AF in the univariate analysis. Comparison of the baseline values of L-arginine and dimethylarginines with the follow-up levels revealed that SDMA was stable over time whereas there was a less substantial relation for ADMA between the two time points. No significant correlation between follow-up and initial values could be observed for L-arginine. Thus, ADMA and L-arginine seem less suitable as long-term biomarkers in contrast to SDMA. Indeed, SDMA proved to be significantly higher in AF- than in ESUS-patients at least one year after stroke. Patients with baseline SDMA values below the calculated cutoff at 0.515 µmol/l were diagnosed only in 8.3% having AF within the total study period. In contrast, 57% of the patients exhibiting SDMA values above this cutoff revealed AF on follow-up. However, like in the initial study, we failed to reveal a clear independency from thrombembolic risk according to the regression analysis. As meanwhile confirmed by several studies, ESUS-patients exhibit significantly less thrombembolic risk factors than stroke patients with determined etiology^23–25^ – an observation that proves true in our collective.

The importance of dimethylarginines in AF and other etiologies of stroke is underscored by other current publications. E.g., Cordts *et al*. recently impressively showed that ratios of L-homoarginine and ADMA or SDMA were associated with stroke etiology and risk prediction in three independent stroke cohorts^26^. Horowitz *et al*. demonstrated that ADMA and SDMA-levels are associated with adverse events and death in anticoagulated AF-patients^27^. Interestingly, a current study of Emrich *et al*. highlighted SDMA as independent biomarker for progression of chronic kidney disease and atherosclerotic vascular events^28^. In accordance to these data, we found significant correlations of dimethylarginines both on follow-up and baseline with thrombembolic risk scores which also proved true within the ESUS-subgroup.

### CIMT as indicator of AF after embolic stroke

CIMT was measured in the course of the initial stroke diagnostics and was identified as independent marker for detection of AF in total as well as newly diagnosed AF. Several CIMT cutoffs proved to be of clinical relevance in our collective as high levels of sensitivity and specificity for detection of AF can be achieved. E.g., at 0.8 mm a clear differentiation of AF-rates over time could be observed in Kaplan-Meier analysis: While in patients with normal CIMT values (<0.8 mm) AF was detected in 14.9% in the whole observation time, in patients with broadened CIMT the rate was 41.9%. Interestingly, in only one patient with CIMT <0.8 mm, AF-diagnosis was made on long-term follow-up. Thus, measurement of CIMT might be useful as future diagnostic tool for stratifying AF risk in ESUS and could therefore support indication for further and prolonged rhythm analyses. These data are supported by several studies which investigated CIMT as AF-indicator in larger samples in the general population^29–31^.

### Novel biomarkers of atrial cardiopathy in ESUS

As described above, current data hint to the assumption that a diseased left atrium may feature enhanced thromboembolic risk before or even autonomously from AF – possibly explaining an additional proportion of strokes in ESUS^7,14,32^. In a subgroup analysis of NAVIGATE ESUS it was recently shown that in ESUS patients with left atrial enlargement anticoagulation might be superior over ASA for secondary prevention^33^. The ARCADIA trial is currently investigating whether a subgroup of ESUS-patients exhibiting features of atrial cardiopathy as determined by elevated NT-proBNP, prolonged PTFV1 or left atrial enlargement might benefit from oral anticoagulation rather than platelet aggregation inhibition^34^. In ARCADIA, left atrial enlargement is defined as left atrial diameter index of ≥3 cm/m^2^ on echocardiogram, which is considered being “severely enlarged”^35^. Likewise, a German consensus paper on the detection of AF in stroke patients stated that AF-probability is comparatively high when left atrial diameter is >45 mm^36^. Remarkably, a recent study by Jalini *et al*. revealed that in only 5.1% of ESUS-patients severe left atrial enlargement could be found^37^. However, this proportion was also relatively low even in patients suffering from diagnosed cardioembolic stroke (25%)^37^. This raises the question whether the high cutoffs of left atrial dimension are appropriate for diagnosis of atrial cardiopathy in stroke patients. Furthermore, atrial disease seems to develop over time and is more advanced and prevalent in AF patients^38^. The association between LAVI and cardioembolic stroke has recently been confirmed in a large study by Jordan *et al*.^39^.

In our current study, we show for the first time that SDMA plasma levels are positively correlated with LAVI in patients with stroke of different etiologies, but – even more important – also in the subgroup of patients who suffered ESUS. These associations were present after adjusting for CHA_2_DS_2_VASC and renal function in the linear regression analysis. Of note, even further associations have been found between SDMA and total atrial conduction time and strain rates, although these did not proof to sustain after Bonferroni correction or in linear regression, respectively.

As stated above, SDMA levels were significantly higher in AF than in ESUS patients. However, this difference was not independent from thrombembolic risk factors. Thus, one could imagine that an early step of atrial cardiopathy may be initially driven by endothelial dysfunction mechanisms (depicted by SDMA). Over time and forced by cardiovascular risk factors, endothelial damage and atherosclerosis progress (depicted by CIMT). Further atrial remodeling processes might result in occurrence of AF - at least in a part of patients with atrial cardiopathy. The question remains, at which point in this longitudinal disease process the effect of an oral anticoagulation (OAC) may become superior to platelet inhibition. However, SDMA and CIMT might be useful to depict this process on a biomarker level.

### Limitations

There are several limitations of this study. Patients were well and deeply characterized, however the total number of patients is relatively small and the findings clearly need to be proven in larger samples before there might be a translation into clinical application. Importantly, there were no additional standardized rhythm analyses conducted on follow-up, but only documentation of the clinically applied Holter-ECG and event recorder analyses. In median, all patients without known AF were provided with a Holter-ECG recording over 72 hours within the whole observation time which did not differ between study groups but might underestimate AF diagnoses as suggested by several studies on cardiac monitoring in stroke patients (for comprehensive review see:^40^). Indeed, in 25% of patients who were initially admitted with ESUS, AF was detected within the whole study period. In comparison, in patients who were enrolled in the CRYSTAL AF trial and besides that fulfilled the criteria of RESPECT-ESUS or NAVIGATE-ESUS the AF detection rate at three years was about 35% using implantable cardiac monitors^41^. Thus, further follow-up studies with longer rhythm analyses or application of implantable loop recorders are clearly warranted to validate our findings.

## Conclusions

In conclusion, our study confirmed CIMT and SDMA as potential markers of AF in patients who suffered embolic stroke. Moreover, SDMA proved to be stable over time and is independently associated with left atrial volume, which is regarded as marker of atrial cardiopathy. These results may support biomarker-based diagnosis of etiology in embolic stroke.

## Methods

### Study population

This is a long-term follow-up study investigating a patient collective that has been prospectively recruited between August 2016 and April 2017 at the Department of Neurology at Hannover Medical School^10,11^. In this study, patients were assigned to four groups according to stroke etiology: (1) embolic stroke of undetermined source (ESUS), (2) cardioembolic stroke due to atrial fibrillation, (3) macroangiopathic stroke and (4) microangiopathic stroke. The ESUS-group was defined according to the following criteria: Detection of an acute ischemic stroke of unknown origin with an embolic pattern, i.e. maximum diameter of at least 2 cm in the subcortical space or with cortical involvement as assessed with diffusion-weighted imaging (DWI)-magnetic resonance imaging (MRI) or at least 1,5 cm in cranial CT or with a cortical correlated lesion. At the same time, patients with atrial fibrillation or a ≥50% stenosis of a vessel supplying the affected brain area were allocated to other groups according to the TOAST criteria^42^.

The previous study contained standardized stroke diagnostics for all patients, including imaging (MRI or CT, MRI- or CT-angiography), Doppler- and duplex ultrasound of the brain supplying arteries and comprehensive echocardiography. Besides standard parameters, echocardiography obtained left atrial volume index (LAVI), septal (s) and lateral (l) total atrial conduction time (PA-TDI) as determined using tissue Doppler imaging and left atrial strain rates, as previously described^10^.

The carotid intima-media thickness (CIMT) was measured in the course of Duplex sonography in both common carotid arteries and amounted to the average mean value. Moreover, in the course of the previous study, all patients in whom no AF was previously known or detected on admission received an additional long-term (Holter-) ECG for a targeted duration of 72 h during in hospital stay. Serum creatinine levels and the estimated glomerular filtration rate (eGFR), computed with the CKD-EPI formula, were available for all patients on admission.

Rare stroke etiologies, such as endocarditis, vasculitis or dissection of the brain supplying arteries or the detection of a deep vein thrombosis (DVT) in combination with patent foramen ovale (PFO) led to the exclusion from the study. The same applied for detection of a current malignant comorbidity. All procedures have been done blinded to the patients’ characteristics.

The ethics committee at Hannover Medical School consented to the extension of the previous study to this long-term follow-up investigation. All patients provided additional written informed consent before being enrolled in the follow-up study. The study was conducted in accordance with the Declaration of Helsinki.

### Clinical evaluation

The follow-up consisted of a standardized interview including the following items: Assessment of demographic data, i.e. sex, age, height, weight, body mass index (BMI), pre-existing conditions, thrombembolic risk factors, recurrent cardio-/cerebrovascular events, current medication and additional cardiac monitoring within the follow-up period as well as the date of newly diagnosed AF, if occurred. The Essen Stroke Risk Score (ESRS) and the CHA_2_DS_2_VASC Score were evaluated for both the initial and the follow-up time point. To quantify stroke severity and outcome, the National Institutes of Health Stroke Scale (NIHSS), the modified Rankin scale (mRS) and the Barthel Index (BI) were used. If further Holter-ECG were performed within the follow-up period, both the duration of this additional monitoring and the duration of Holter-ECG in the previous study were subsumed as cumulative duration. Peripheral venous blood was obtained from each patient visited in person. If no on-site visit could be conducted, the mRS, CHA_2_DS_2_VASC, ESRS, BI and clinical data were obtained via phone.

### Biomarker analysis

At follow-up on-site examination peripheral venous blood was taken from all patients visited. Blood was centrifuged for 15 minutes at 1.600 × g. EDTA plasma samples were stored until analysis at −80 °C. Levels of L-arginine, ADMA and SDMA were detected using high-performance liquid chromatography–tandem mass spectrometry (HPLC-MS-MS)^43^ at the Institute of Clinical Pharmacology at Magdeburg University.

### Statistical analysis

The statistical analysis was done using IBM SPSS Statistics 25 (SPSS Inc., Chicago, IL. USA). Distribution of data was tested using the Kolmogorov-Smirnov test. Group differences were detected using the Student’s t-test or Mann-Whitney-U-test, as appropriate. For categorically distributed data, the chi-square test was applied. ANOVA or the Kruskal-Wallis-test were used for a comparison of more than two groups, as appropriate. Correlations were calculated according to the Spearman rho test or Pearson correlation. Bonferroni correction applied for multiple testing. Linear regression analysis was done including relevant baseline characteristics. Accordingly, in dichotomized comparisons the binary logistic regression analysis was executed, using the backward stepwise calculation method. A p-value of <0.05 was considered significant. ROC-analysis was conducted to analyze the ability of markers to discriminate between ESUS and AF or ESUS and newly diagnosed AF, including the corresponding area under the curve (AUC). The Youden-index was calculated to determine optimal cut-offs. Cumulative AF-detection rate was estimated by the Kaplan-Meier method and compared via log-rank test between dichotomized marker levels for CIMT and SDMA. Figures were created using GraphPad Prism 5 (GraphPad Inc., La Jolla, CA, USA) and IBM SPSS Statistics 25 (SPSS Inc., Chicago, IL. USA).

## Supplementary information

Supplemental Information

## Data Availability

The datasets generated during and analyzed during the current study are available from the corresponding author on reasonable request.

## References

[1] Hart RG (2014). Embolic strokes of undetermined source: the case for a new clinical construct. Lancet Neurol..

[2] Hart RG (2016). Rivaroxaban for secondary stroke prevention in patients with embolic strokes of undetermined source: Design of the NAVIGATE ESUS randomized trial. European Stroke Journal.

[3] Diener HC (2015). Design of Randomized, double-blind, Evaluation in secondary Stroke Prevention comparing the EfficaCy and safety of the oral Thrombin inhibitor dabigatran etexilate vs. acetylsalicylic acid in patients with Embolic Stroke of Undetermined Source (RE-SPECT ESUS). Int. J. Stroke.

[4] Hart RG (2018). Rivaroxaban for Stroke Prevention after Embolic Stroke of Undetermined Source. N. Engl. J. Med..

[5] Diener HC (2019). Dabigatran for Prevention of Stroke after Embolic Stroke of Undetermined Source. N. Engl. J. Med..

[6] Elkind MSV (2018). Atrial Cardiopathy and Stroke Prevention. Curr. Cardiol. Rep..

[7] Yaghi S, Kamel H, Elkind MSV (2017). Atrial cardiopathy: a mechanism of cryptogenic stroke. Expert Rev. Cardiovasc. Ther..

[8] Wijesurendra RS, Casadei B (2015). Atrial fibrillation: effects beyond the atrium?. Cardiovasc. Res..

[9] Willeit K, Kiechl S (2014). Atherosclerosis and atrial fibrillation–two closely intertwined diseases. Atherosclerosis.

[10] Sieweke, J. T. *et al*. Septal total atrial conduction time for prediction of atrial fibrillation in embolic stroke of unknown source: a pilot study. *Clin. Res. Cardiol*. (2019). 10.1007/s00392-019-01501-2 PMC6989646 31236691

[11] Grosse, G. M. *et al*. Plasma Dimethylarginine Levels and Carotid Intima-Media Thickness are related to Atrial Fibrillation in Patients with Embolic Stroke. *Int. J. Mol. Sci*. **20**, 730 (2019). 10.3390/ijms20030730 PMC6387438 30744089

[12] Ramuschkat M (2016). ADMA, subclinical changes and atrial fibrillation in the general population. Int. J. Cardiol..

[13] Sattler, K. *et al*. Occlusion of left atrial appendage affects metabolomic profile: focus on glycolysis, tricarboxylic acid and urea metabolism. *Metabolomics* **13**, 127-017–1255-2. Epub 2017 Sep 20 (2017). 10.1007/s11306-017-1255-2 PMC5772135 29391863

[14] Kamel H (2015). Electrocardiographic Left Atrial Abnormality and Risk of Stroke: Northern Manhattan Study. Stroke.

[15] Goda T (2017). P-Wave Terminal Force in Lead V1 Predicts Paroxysmal Atrial Fibrillation in Acute Ischemic Stroke. J. Stroke Cerebrovasc Dis..

[16] Adami, A. *et al*. Electrocardiographic RR Interval Dynamic Analysis to Identify Acute Stroke Patients at High Risk for Atrial Fibrillation Episodes During Stroke Unit Admission. *Transl. Stroke Res*. (2018). 10.1007/s12975-018-0645-8 PMC6526141 29971705

[17] Sebasigari D (2017). Biomarkers of Atrial Cardiopathy and Atrial Fibrillation Detection on Mobile Outpatient Continuous Telemetry After Embolic Stroke of Undetermined Source. J. Stroke Cerebrovasc Dis..

[18] Montaner J (2008). Etiologic diagnosis of ischemic stroke subtypes with plasma biomarkers. Stroke.

[19] Seegers, J. *et al*. Natriuretic peptides for the detection of paroxysmal atrial fibrillation. *Open Heart* **2**, e000182–2014-000182. eCollection 2015 (2015). 10.1136/openhrt-2014-000182 PMC4533200 26288739

[20] Llombart V (2015). B-type natriuretic peptides help in cardioembolic stroke diagnosis: pooled data meta-analysis. Stroke.

[21] Jensen JK (2006). Serial measurements of N-terminal pro-brain natriuretic peptide after acute ischemic stroke. Cerebrovasc. Dis..

[22] Yip HK (2006). Time course and prognostic value of plasma levels of N-terminal pro-brain natriuretic peptide in patients after ischemic stroke. Circ. J..

[23] Kasner, S. E. *et al*. Characterization of Patients with Embolic Strokes of Undetermined Source in the NAVIGATE ESUS Randomized Trial. *J. Stroke Cerebrovasc Dis*. (2018). 10.1016/j.jstrokecerebrovasdis.2018.01.027 PMC6701183 29525076

[24] Hawkes MA (2018). Differential characteristics, stroke recurrence, and predictors of covert atrial fibrillation of embolic strokes of undetermined source. Int. J. Stroke.

[25] Perera KS (2016). Embolic strokes of undetermined source: Prevalence and patient features in the ESUS Global Registry. Int. J. Stroke.

[26] Cordts K (2018). Guanidino compound ratios are associated with stroke etiology, internal carotid artery stenosis and CHA2DS2-VASc score in three cross-sectional studies. J. Neurol. Sci..

[27] Horowitz JD (2018). Asymmetric and Symmetric Dimethylarginine Predict Outcomes in Patients With Atrial Fibrillation: An ARISTOTLE Substudy. J. Am. Coll. Cardiol..

[28] Emrich IE (2018). Symmetric dimethylarginine (SDMA) outperforms asymmetric dimethylarginine (ADMA) and other methylarginines as predictor of renal and cardiovascular outcome in non-dialysis chronic kidney disease. Clin. Res. Cardiol..

[29] Adamsson Eryd S (2014). Carotid intima-media thickness is associated with incidence of hospitalized atrial fibrillation. Atherosclerosis.

[30] Proietti M (2015). Relationship between carotid intima-media thickness and non valvular atrial fibrillation type. Atherosclerosis.

[31] Chen, L. Y. *et al*. Carotid Intima-Media Thickness and Arterial Stiffness and the Risk of Atrial Fibrillation: The Atherosclerosis Risk in Communities (ARIC) Study, Multi-Ethnic Study of Atherosclerosis (MESA), and the Rotterdam Study. *J. Am. Heart Assoc*. **5 **(2016). 10.1161/JAHA.115.002907 PMC4889172 27207996

[32] Kamel H (2018). Atrial Cardiopathy and the Risk of Ischemic Stroke in the CHS (Cardiovascular Health Study). Stroke.

[33] Healey, J. S. *et al*. Recurrent Stroke With Rivaroxaban Compared With Aspirin According to Predictors of Atrial Fibrillation: Secondary Analysis of the NAVIGATE ESUS Randomized Clinical Trial. *JAMA Neurol* (2019). 10.1001/jamaneurol.2019.0617 PMC6583060 30958508

[34] Kamel H (2019). The AtRial Cardiopathy and Antithrombotic Drugs In prevention After cryptogenic stroke randomized trial: Rationale and methods. Int. J. Stroke.

[35] Lang RM (2006). Recommendations for chamber quantification. Eur. J. Echocardiogr..

[36] Haeusler KG (2018). Expert opinion paper on atrial fibrillation detection after ischemic stroke. Clin. Res. Cardiol..

[37] Jalini, S. *et al*. Atrial cardiopathy in patients with embolic strokes of unknown source and other stroke etiologies. *Neurology* (2018). 10.1212/WNL.0000000000006748 30518556

[38] Leifer, D. & Rundek, T. Atrial cardiopathy: A new cause for stroke? *Neurology* (2018). 10.1212/WNL.0000000000006749 30518553

[39] Jordan, K. *et al*. Left Atrial Volume Index Is Associated With Cardioembolic Stroke and Atrial Fibrillation Detection After Embolic Stroke of Undetermined Source. *Stroke*, STROKEAHA119025384 (2019). 10.1161/STROKEAHA.119.025384 PMC6646078 31189435

[40] Haeusler KG (2018). Detection of Atrial Fibrillation in Cryptogenic Stroke. Curr. Neurol. Neurosci. Rep..

[41] Verma, N. *et al.* Incidence of atrial fibrillation among patients with an embolic stroke of undetermined source: Insights from insertable cardiac monitors. *Int. J. Stroke*, 1747493018798554 (2018). 10.1177/1747493018798554 30196791

[42] Adams HP (1993). Classification of subtype of acute ischemic stroke. Definitions for use in a multicenter clinical trial. TOAST. Trial of Org 10172 in Acute Stroke Treatment. Stroke.

[43] Martens-Lobenhoffer J, Bode-Boger SM (2012). Quantification of L-arginine, asymmetric dimethylarginine and symmetric dimethylarginine in human plasma: a step improvement in precision by stable isotope dilution mass spectrometry. J. Chromatogr. B. Analyt Technol. Biomed. Life. Sci..

