# Supplementary material for: Markers of endothelial pathology to support detection of atrial fibrillation in embolic stroke of undetermined source
Source: Sci Rep. 2019 Dec 19;9:19424. doi: 10.1038/s41598-019-55943-9 (PMC6923420; doi:10.1038/s41598-019-55943-9)

**Supplemental material**

**Markers of endothelial pathology to support detection of atrial fibrillation in embolic stroke of undetermined source**

Nora L. Ziegler^A^, Jan-Thorben Sieweke, MD^B^, Saskia Biber^A,B^, Maria M. Gabriel^A^, Ramona Schuppner, MD^A^, Hans Worthmann, MD^A^, Jens Martens-Lobenhoffer, PhD^C^, Ralf Lichtinghagen, PhD^D^, Stefanie M. Bode-Böger, MD^C^, Udo Bavendiek, MD^B^, Karin Weissenborn, MD^A^, Gerrit M. Grosse, MD^A*^

^A^Department of Neurology, Hannover Medical School, Hannover, Germany

^B^Department of Cardiology and Angiology, Hannover Medical School, Hannover, Germany

^C^Institute of Clinical Pharmacology, Otto-von-Guericke University Magdeburg,

Magdeburg, Germany

^D^Institute of Clinical Chemistry, Hannover Medical School, Hannover, Germany

*Karin Weissenborn and Gerrit M. Grosse contributed equally

Appropriate therapy and medication intake

For an overview of secondary stroke preventive therapy see Table S1. An oral anticoagulation using non-Vitamin K oral anticoagulants (NOAC) or vitamin K antagonists (VKA) was performed in 26 individuals, while 73 patients were treated with ASA for secondary prevention. Six subjects obtained dual platelet inhibition. 78 patients took statins, 29 received angiotensin converting enzyme (ACE) inhibitors and 36 angiotensin II receptor blockers (ARBs), beta-adrenoreceptor blocking drugs were administered to 38 patients and 19 took vitamin supplements.

94 patients stated a regular intake of the prescribed medication, one patient insisted on continuing to take aspirin instead of anticoagulative therapy despite being diagnosed with AF. Accordingly, five of 98 patients did not receive an appropriate therapy. Two of these patients suffered new ischemic events, both belonging to the ESUS-group.

Recurrent cardio- or cerebrovascular events

18 patients suffered at least one secondary cerebro- or cardiovascular ischemic event. 10 patients had a new stroke and 8 had one or more TIA during the follow-up period. This affected 7 patients (15.56%) in the ESUS-group, 6 in the group with AF (26.09%), three patients with macro- (27.27%) and two patients with microangiopathic stroke (10.53%). One patient suffered a myocardial infarction within the follow-up period in addition to another recurrent stroke. Moreover, two patients were treated for acute coronary syndrome. Another patient suffered a deep vein thrombosis during the follow-up period. According to chi-square test there were no significant differences between the study groups concerning cardio- or cerebrovascular recurrent events.

Markers of endothelial pathology and thrombembolic risk

SDMA levels on follow-up were positively correlated with ESRS (r=0.322, P=0.002) and CHA_2_DS_2_VASC (r=0.362, P=0.001) after Bonferroni-correction in the whole study collective (Table S4). In the ESUS-subgroup, SDMA levels on follow-up proved to be correlated with CHA_2_DS_2_VASC (r=0.401, P=0.008), but not with ESRS (r=0.307, P=0.048), after Bonferroni-correction. There was a positive correlation of follow-up ADMA levels with CHA_2_DS_2_VASC considering the ESUS-subgroup (r=0.423, P=0.005, see Table S5). However, neither SDMA nor ADMA levels on follow-up were associated with recurrent ischemic events in the whole study collective or in the ESUS-subgroup.

By contrast, CIMT was significantly broadened in patients with recurrent ischemic events in the total cohort compared to those without suffering new events (P=0.009). In the binary logistic regression analysis, this difference was independent from thrombembolic risk as estimated with CHA_2_DS_2_VASC or ESRS (P=0.046, P=0.025, respectively). In the ESUS-subgroup, there was a non-significant difference regarding CIMT values between patients with and without recurrent ischemic events (P=0.077). Likewise, after Bonferroni-correction CIMT showed no significant correlation with ESRS (r= 0.259; P=0.086) or CHA_2_DS_2_VASC (r=0.377; P=0.011) in ESUS patients. However, in the total sample, CIMT was closely associated with CHA_2_DS_2_VASC and ESRS (r=0.443; P<0.001 and r=0.421; P<0.001, respectively, see Table S4).

**Supplemental Tables**

**Supplemental Table I:** Secondary stroke preventive therapy

|  | **ESUS** | **AF**  **(total)** | **Macroangiopathic**  **stroke** | **Microangiopathic stroke** | **P-value** |
| --- | --- | --- | --- | --- | --- |
| N | 45 | 23 | 11 | 19 |  |
| ASA (N = 73) | 41  (91.11%) | 2  (8.70%) | 11  (100.00%) | 19  (100.00%) | < 0.001 |
| NOAC (N = 23) | 2  (4.44%) | 21  (91.30%) | 0  (0.00%) | 0  (0.00%) | < 0.001 |
| VKA (N = 3) | 1  (2.22%) | 1  (4.35%) | 0  (0.00%) | 1  (5.26%) | 0.827 |
| Dual platelet inhibition  (N = 6) | 0  (0.00%) | 0  (0.00%) | 5  (45.45%) | 1  (5.26%) | < 0.001 |
| Statins (N = 78) | 33  (73.33%) | 19  (82.61%) | 9  (81.82%) | 17  (89.47%) | 0.496 |
| ACE inhibitors  (N = 29) | 10  (22.22%) | 11  (47.83%) | 3  (27.27%) | 5  (26.32%) | 0.174 |
| ARBs (N = 36) | 15  (33.33%) | 9  (39.13%) | 4  (36.36%) | 8  (42.11%) | 0.915 |
| Betaadrenoceptor blocking drugs (N = 38) | 14  (31.11%) | 14  (60.87%) | 4  (36.36%) | 6  (31.58%) | 0.099 |
| Vitamin supplements  (N = 19) | 9  (20.00%) | 4  (17.39%) | 0  (0.00%) | 6  (31.58%) | 0.210 |

P-values were calculated using chi-square test. A p-value < 0.05 was considered significant.

**Supplemental Table II:** Dimethylarginines and echocardiographic parameters of the left atrium (whole study collective)

|  |  | L-arginine baseline | ADMA baseline | SDMA baseline | L-arginine follow-up | ADMA follow-up | SDMA follow-up | CIMT |
| --- | --- | --- | --- | --- | --- | --- | --- | --- |
| **LAVI** | **r** | - 0.242 | 0.145 | 0.313^*^ | 0.103 | 0.397^**^ | 0.322^**^ | 0.211^*^ |
|  | **p-value** | 0.064 | 0.274 | **0.016** | 0.356 | **< 0.001** | **0.003** | **0.041** |
|  | **n** | 59 | 59 | 59 | 83 | 83 | 83 | 94 |
| **PA-TDI septal** | **r** | - 0.299^*^ | - 0.144 | 0.338^**^ | - 0.162 | 0.068 | 0.203 | 0.139 |
|  | **p-value** | **0.022** | 0.282 | **0.009** | 0.145 | 0.546 | 0.067 | 0.185 |
|  | **n** | 58 | 58 | 58 | 82 | 82 | 82 | 93 |
| **PA-TDI lateral** | **r** | - 0.300^*^ | - 0.003 | 0.283^*^ | - 0.232^*^ | 0.140 | 0.203 | 0.088 |
|  | **p-value** | **0.022** | 0.982 | **0.031** | **0.036** | 0.211 | 0.068 | 0.403 |
|  | **n** | 58 | 58 | 58 | 82 | 82 | 82 | 93 |
| **LA GLS** | **r** | 0.259 | 0.030 | - 0.333^*^ | 0.011 | - 0.178 | - 0.203 | - 0.263^*^ |
|  | **p-value** | 0.057 | 0.825 | **0.013** | 0.926 | 0.116 | 0.072 | **0.012** |
|  | **n** | 55 | 55 | 55 | 79 | 79 | 79 | 90 |
| **SRs** | **r** | 0.254 | 0.039 | - 0.208 | 0.043 | - 0.073 | - 0.225^*^ | - 0.097 |
|  | **p-value** | 0.056 | 0.773 | 0.120 | 0.705 | 0.519 | **0.043** | 0.359 |
|  | **n** | 57 | 57 | 57 | 81 | 81 | 81 | 92 |
| **SRe** | **r** | - 0.222 | 0.189 | 0.321^*^ | - 0.064 | 0.077 | 0.174 | 0.386^**^ |
|  | **p-value** | 0.098 | 0.159 | **0.015** | 0.569 | 0.497 | 0.121 | **< 0.001** |
|  | **n** | 57 | 57 | 57 | 81 | 81 | 81 | 92 |
| **SRa** | **r** | - 0.216 | 0.050 | 0.269^*^ | - 0.102 | 0.218 | 0.224^*^ | 0.103 |
|  | **p-value** | 0.106 | 0.714 | **0.043** | 0.365 | **0.050** | **0.045** | 0.330 |
|  | **n** | 57 | 57 | 57 | 81 | 81 | 81 | 92 |
| **LAVI / A‘** | **r** | - 0.225 | 0.166 | 0.378^**^ | - 0.063 | 0.284^**^ | 0.374^**^ | 0.253^*^ |
|  | **p-value** | 0.089 | 0.212 | **0.003** | 0.573 | **0.010** | **0.001** | **0.014** |
|  | **n** | 58 | 58 | 58 | 82 | 82 | 82 | 93 |

Values were calculated using Spearman correlation. P-values <0.05 are highlighted in bold letters. A p-value <0.006 was considered significant after Bonferroni correction, accounting for echocardiography-parameters (bold and underlined).

**Supplemental Table III:** Dimethylarginines and echocardiographic parameters of the left atrium (ESUS subgroup)

|  |  | L-arginine baseline | ADMA baseline | SDMA baseline | L-arginine follow-up | ADMA follow-up | SDMA follow-up | CIMT |
| --- | --- | --- | --- | --- | --- | --- | --- | --- |
| **LAVI** | **r** | - 0.144 | 0.177 | 0.376 | 0.219 | 0.563 | 0.446 | 0.271 |
|  | **p-value** | 0.422 | 0.324 | **0.031** | 0.169 | **< 0.001** | **0.003** | 0.075 |
|  | **n** | 33 | 33 | 33 | 41 | 41 | 41 | 44 |
| **PA-TDI septal** | **r** | - 0.076 | - 0.072 | 0.275 | 0.046 | 0.107 | 0.294 | 0.15 |
|  | **p-value** | 0.675 | 0.691 | 0.121 | 0.776 | 0.507 | 0.062 | 0.33 |
|  | **n** | 33 | 33 | 33 | 41 | 41 | 41 | 44 |
| **PA-TDI lateral** | **r** | - 0.329 | - 0.08 | 0.336 | - 0.136 | 0.283 | 0.426 | 0.002 |
|  | **p-value** | 0.062 | 0.659 | 0.056 | 0.396 | 0.073 | **0.005** | 0.988 |
|  | **n** | 33 | 33 | 33 | 41 | 41 | 41 | 44 |
| **LA GLS** | **r** | 0.324 | 0.203 | - 0.013 | - 0.175 | - 0.229 | - 0.118 | - 0.129 |
|  | **p-value** | 0.075 | 0.272 | 0.946 | 0.287 | 0.161 | 0.475 | 0.417 |
|  | **n** | 31 | 31 | 31 | 39 | 39 | 39 | 42 |
| **SRs** | **r** | 0.191 | 0.106 | - 0.159 | - 0.07 | - 0.242 | - 0.394 | - 0.126 |
|  | **p-value** | 0.294 | 0.564 | 0.386 | 0.669 | 0.133 | **0.012** | 0.421 |
|  | **n** | 32 | 32 | 32 | 40 | 40 | 40 | 43 |
| **Sre** | **r** | - 0.358 | 0.125 | 0.388 | - 0.034 | 0.279 | 0.158 | 0.395 |
|  | **p-value** | **0.044** | 0.494 | **0.028** | 0.833 | 0.082 | 0.33 | **0.009** |
|  | **n** | 32 | 32 | 32 | 40 | 40 | 40 | 43 |
| **Sra** | **r** | - 0.252 | - 0.03 | 0.117 | 0.012 | 0.19 | 0.245 | - 0.043 |
|  | **p-value** | 0.164 | 0.872 | 0.525 | 0.941 | 0.24 | 0.128 | 0.785 |
|  | **n** | 32 | 32 | 32 | 40 | 40 | 40 | 43 |
| **LAVI / A‘** | **r** | - 0.187 | 0.134 | 0.312 | 0.025 | 0.384 | 0.353 | 0.08 |
|  | **p-value** | 0.298 | 0.459 | 0.078 | 0.878 | **0.013** | **0.024** | 0.608 |
|  | **n** | 33 | 33 | 33 | 41 | 41 | 41 | 44 |

Values were calculated using Spearman correlation. P-values <0.05 are highlighted in bold letters. A p-value <0.006 was considered significant after Bonferroni correction, accounting for echocardiography-parameters (bold and underlined).

**Supplemental Table IV:** Markers of endothelial pathology and thrombembolic risk (whole study collective)

|  |  | L-arginine baseline | ADMA baseline | SDMA baseline | L-arginine follow-up | ADMA follow-up | SDMA follow-up | CIMT |
| --- | --- | --- | --- | --- | --- | --- | --- | --- |
| CHA_2_DS_2_VASC | r | - 0.271^*^ | 0.160 | 0.415^**^ | - 0.064 | 0.249^*^ | 0.362^**^ | 0.443^**^ |
|  | P-Value | **0.032** | 0.210 | **0.001** | 0.555 | **0.020** | **0.001** | **< 0.001** |
|  | N | 63 | 63 | 63 | 87 | 87 | 87 | 98 |
| ESRS | r | - 0.286^*^ | 0.190 | 0.538^**^ | -0.061 | 0.211^*^ | 0.322^**^ | 0.421^**^ |
|  | P-Value | **0.023** | 0.136 | **< 0.001** | 0.574 | 0.050 | **0.002** | **< 0.001** |
|  | N | 63 | 63 | 63 | 87 | 87 | 87 | 98 |

Values were calculated using Spearman correlation. P-values <0.05 are highlighted in bold letters. A p-value <0.025 was considered significant after Bonferroni correction accounting for the two risk scores (bold and underlined).

**Supplemental Table V:** Dimethylarginines and thrombembolic risk (ESUS-subgroup)

|  |  | L-arginine baseline | ADMA baseline | SDMA baseline | L-arginine follow-up | ADMA follow-up | SDMA follow-up | CIMT |
| --- | --- | --- | --- | --- | --- | --- | --- | --- |
| CHA_2_DS_2_VASC | r | -0.516^**^ | 0.138 | 0.301 | - 0.081 | 0.423^**^ | 0.401^**^ | .377^*^ |
|  | P-Value | **0.002** | 0.436 | 0.084 | 0.610 | **0.005** | **0.008** | **0.011** |
|  | N | 34 | 34 | 34 | 42 | 42 | 42 | 45 |
| ESRS | r | - 0.483^**^ | 0.246 | 0.498^**^ | - 0.060 | 0.361^*^ | 0.307^*^ | 0.259 |
|  | P-Value | **0.004** | 0.160 | **0.003** | 0.704 | **0.019** | **0.048** | 0.086 |
|  | N | 34 | 34 | 34 | 42 | 42 | 42 | 45 |

Values were calculated using Spearman correlation. P-values <0.05 are highlighted in bold letters. A p-value <0.01 was considered significant after Bonferroni-correction accounting for the two risk scores (bold and underlined).

**Supplemental Figure I:** Overview of patients’ recruitment.


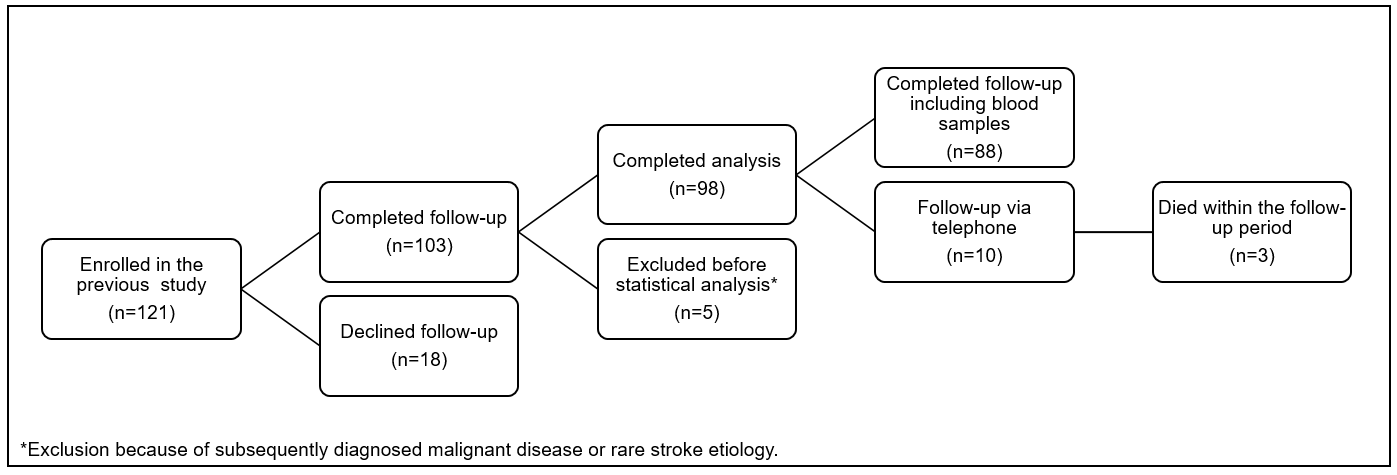

Supplement: Supplementary file 1 — Supplemental Information [file 41598_2019_55943_MOESM1_ESM.docx]
